# Threshold effects of interactional context on attentional control

**DOI:** 10.3389/fpsyg.2026.1836643

**Published:** 2026-05-29

**Authors:** Min Li, Qinghong Xu, Xiangyu Shi, Shiwen Zhang, Jie Li

**Affiliations:** 1 School of Psychology, Inner Mongolia Normal University, Hohhot, China; 2 School of Education, Inner Mongolia Minzu University, Tongliao, China; 3 School of Foreign Languages, Yulin University, Yulin, China; 4 Student Affairs Department, Wuhai Vocational and Technical College, Wuhai, China; 5 School of Management, Tianjin University of Traditional Chinese Medicine, Tianjin, China

**Keywords:** adaptive, attentional control, cognitive control, interactional context, language switching

## Abstract

**Introduction:**

The debate surrounding bilingual cognitive advantages has prompted researchers to focus on the dynamic regulatory mechanisms of bilingual experience.

**Methods:**

Using a cross-task priming paradigm, the present study investigated the threshold effects of bilingual interactional contexts on attentional control. In Experiment 1, 60 unbalanced Chinese-English bilinguals completed a single picture-naming task involving four interactional contexts (monolingual English, monolingual Chinese, bilingual, and voluntary naming). This was followed by an anti-saccade task. The results showed that a single exposure to dual-language contexts did not facilitate attentional control. Instead, it induced resource depletion, as evidenced by significantly prolonged reaction times. In Experiment 2, 40 bilinguals completed two consecutive blocks of the same picture naming task before performing the anti-saccade task.

**Results:**

The results showed that, compared with monolingual contexts, repeated exposure to dual-language contexts significantly enhanced attentional control, as indexed by faster reaction times and higher accuracy. Moreover, the facilitatory effect was stronger in the bilingual context than in the voluntary naming context. The contrast between the two experiments reveals a cumulative threshold of cognitive load: a single exposure falls below the threshold, resulting in resource depletion, whereas repeated exposure surpasses it, triggering adaptive reorganization of attentional control.

**Discussion:**

The present study provides preliminary evidence that the influence of bilingual contexts on attentional control may follow a threshold effect, offering a potential mechanistic account of bilingual cognitive plasticity.

## 1 Introduction

Whether bilingual experience confers advantages in domain-general cognitive control has generated considerable debate. Early studies reported benefits in tasks tapping inhibitory control (Kałamała et al., 2022), task switching (Yang et al., 2018), and working memory (Bialystok et al., 2014), leading to the proposal that managing two languages enhances executive functions. However, meta-analytic reviews have challenged the robustness of these effects, suggesting that bilingual advantages may be restricted to specific conditions or populations (Paap et al., 2015). Rather than attempting to resolve this controversy, the present study focuses on the mechanisms that might underlie the relationship between bilingual experience and cognition. Specifically, we ask: by what process might bilingual interactional contexts modulate attentional control?

### 1.1 Interactional context and cognitive control

The Adaptive Control Hypothesis posits that bilinguals experience distinct cognitive demands across three interactional contexts: monolingual, bilingual, and dense code-switching context (Green and Abutalebi, 2013). In bilingual contexts, bilinguals exhibit higher linguistic control demands for multiple processes. These include goal maintenance, interference suppression, salience cue detection, selective response mechanisms, task engagement, and task disengagement. This occurs because bilinguals must simultaneously monitor the target language, suppress interference from the non-target language, and prepare for timely code-switching between languages. In dense code-switching context, bilinguals exhibit the highest linguistic control demands for opportunistic planning (the ability to utilize vocabulary from any readily accessible language for speech production), where they mix languages within a single utterance to achieve rapid expression. In monolingual contexts, bilinguals require language control only for target maintenance and interference suppression, with overall lower language control demands necessitating only weak monitoring of the non-target language (Green and Abutalebi, 2013). Critically, these contextual demands are hypothesized to engage domain-general attentional control mechanisms. Repeated engagement with a particular context may temporarily tune the attentional system to a state of readiness. This state can carry over to subsequent non-linguistic tasks (Jiao et al., 2020a; Timmer et al., 2019).

Behavioral measurement research methods provide evidence for exploring the relationship between interactional contexts and cognitive control. Hartanto and Yang (2016) employed questionnaires to have bilinguals report the frequency of using a single language vs. both languages in different settings (e.g., home, school, and work). This allowed them to distinguish bilinguals in monolingual vs. bilingual interactional contexts. They found that bilinguals in bilingual contexts demonstrated superior switching abilities compared to those in monolingual contexts, suggesting that the conversational context in bilingual communication moderates task switching. To measure different interactional contexts more precisely, Hartanto and Yang (2020) employed a revised Bilingual Interactional Context Questionnaire. This version added assessments of the percentage of time bilinguals spent in home, school, work, and other settings, while also incorporating evaluations of intensive dense code-switching contexts. They calculated the correlation between three distinct Interactional Context indices and cognitive control. Results indicated that the Interactional Context of bilinguals is a key factor influencing executive function. Bilinguals in bilingual and intensive dense code-switching contexts demonstrated advantages in certain executive functions, such as task switching, inhibitory control, and goal maintenance.

Laboratory-based priming methods explore the impact of interactional contexts on cognitive control. Jiao et al. (2020a) employed alternating picture-naming and Flanker tasks for cross-task adaptation. Participants were instructed to name objects in either Chinese or English, depending on the task requirements, thereby creating a monolingual context. Mixing Chinese and English created a bilingual context (Jiao et al., 2020a). Similar studies included monolingual groups, using the same paradigm to investigate this issue (Chung-Fat-Yim et al., 2021). Liu similarly employed a cross-task paradigm and inter-subject EEG synchrony to examine how language control influences cognitive control across different interactional contexts (Liu et al., 2017). Findings revealed that conflict monitoring and inhibitory control, which are involved in language processing, adaptively extend to general cognitive control. This effect was more pronounced in mixed-language vs. single-L2 contexts, where greater language control demands led to heightened inter-subject synchrony. These results provide evidence that interactional Contexts shape cognitive control.

In summary, studies using behavioral questionnaires typically employ between-subjects designs. This approach can introduce additional error due to individual differences and ambiguous context classification. Moreover, a growing body of research has begun to examine the relationship between different context types and cognitive control, supporting the notion of context-specific modulation (Han et al., 2023; Yang et al., 2023). However, existing research has primarily focused on the differential effects of context types on cognitive control, with little attention paid to how the intensity and cumulativity of contextual exposure shape cognitive outcomes. It remains largely unexplored whether, and to what extent, engaging in dual-language contexts influences cognitive control, and whether such influence would manifest as resource depletion or cognitive gain. The present study addresses these limitations by employing a within-subjects priming design, in which each participant experiences all four interactional contexts. This approach eliminates between-subjects variability and allows for a more precise examination of how contextual exposure modulates attentional control.

### 1.2 From inhibitory control to attentional control: a theoretical shift

Addressing this question first requires clarifying the core mechanism through which bilingual experience shapes cognition. Early theoretical accounts emphasized inhibitory control as the key mechanism underlying bilingual cognitive changes, positing that bilinguals frequently suppress the non-target language, thereby enhancing domain-general inhibitory abilities (Green, 1998). However, this framework struggles to account for findings that bilinguals perform better in both congruent and incongruent trials of conflict tasks; meta-analytic evidence suggests that bilinguals may enjoy a broader executive processing advantage, extending beyond inhibitory control alone (Hilchey and Klein, 2011). As Hilchey and Klein (2011) themselves noted, however, the bilingual advantage on conflict resolution is “sporadic at best, and in some cases conspicuously absent.” Recent perspectives propose attentional control as the core mechanism explaining bilingual cognitive changes. Attentional control refers to the ability to flexibly allocate cognitive resources, maintain goal-relevant information, and filter distraction in the service of goal-directed behavior (Draheim et al., 2022). This framework does not dismiss inhibitory control; rather, it subsumes inhibition as one component within a more comprehensive system encompassing goal maintenance, monitoring, and adaptive resource allocation. According to the Supervisory System model, attentional control comprises multiple component processes, including energizing schemata, inhibiting irrelevant responses, monitoring schema activity, and switching between tasks, rather than a single supervisory system (Stuss et al., 1995). Accordingly, according to this perspective, the purported bilingual cognitive advantage is reframed not merely as interference suppression, but as a more dynamic and adaptive regulation of attentional resources (Bialystok and Craik, 2022).

Crucially, the dynamic nature of attentional control has been further elucidated by the Malleable Attentional Resources Theory (MART; Young and Stanton, 2002), which posits that attentional capacity is not a static reservoir but a system that actively adjusts in response to task demands. According to this view, when cognitive load falls below an individual's regulatory capacity, resources are merely passively consumed, resulting in a temporary decline in task performance. Only when accumulated load exceeds a critical point does the system initiate resource reorganization and expansion. This forms a more efficient attentional control system that subsequently yields transfer effects to ensuing tasks. This theoretical framework provides a mechanistic account for how sustained cognitive engagement can lead to adaptive changes in attentional control. Applied to the bilingual context, this perspective suggests that the intensity and cumulativity of exposure to dual-language contexts may determine whether attentional control is merely depleted or adaptively enhanced. This focus on intensity and cumulativity is central to our theoretical model. Previous research has primarily compared different types of contexts without considering how the dose or duration of exposure shapes cognitive outcomes. Although, a single exposure has been found to modulate executive control at the neural level (Jiao et al., 2020a), corresponding behavioral effects were not observed. The present study therefore aimed to investigate under what behavioral conditions such effects would emerge. The distinction between single vs. repeated exposure is critical because it allows us to test the threshold prediction. A single exposure may fall below the threshold, but its effect on attentional control (if any) is unknown. In contrast, repeated exposure may accumulate load beyond the threshold and trigger adaptive reorganization of attentional control. Several lines of evidence support the view that attentional control provides a better explanation than inhibitory control for bilingual cognitive changes. This evidence comes from two aspects. First, bilinguals consistently outperform monolinguals in conflict tasks under congruent conditions. According to traditional explanations of inhibitory mechanisms, if bilinguals frequently train their inhibitory abilities through language switching, they should excel in interference tasks (incongruent conditions). However, empirical studies reveal that bilinguals outperform monolinguals in both congruent and incongruent conditions (Hilchey and Klein, 2011). Furthermore, bilinguals exhibit greater N450 amplitude in non-conflict Stroop trials across both languages, but not in conflict Stroop trials. This suggests that bilinguals' potential advantage may not lie in conflict-specific control, but rather in more general cognitive control (Coderre and Van Heuven, 2014).

On the other hand, tasks examining inhibitory components have less frequently revealed bilingual cognitive changes. Previous studies typically employed the N-back task to assess working memory updating, task switching paradigms to operationalize cognitive flexibility, Stroop, Flanker, and Simon tasks to evaluate interference inhibition, and Go/Nogo tasks to assess response inhibition. Researchers aim to identify the specific components and tasks where bilingual cognitive changes occur. For instance, bilinguals have been reported to demonstrate superior performance in N-back tasks (Comishen and Bialystok, 2021), while fewer cognitive alterations are observed in Simon and Flanker tasks (Costa et al., 2008; Linck et al., 2008). Both aspects of this question are difficult to fully explain based on inhibitory mechanisms alone. They also indirectly suggest that language use in bilinguals may strengthen global monitoring abilities.

Direct evidence for the cognitive changes associated with bilingualism comes from studies examining attentional processes in bilinguals vs. monolinguals. Using a cue-target paradigm, Hernández et al. (2012) investigated whether bilinguals demonstrate enhanced attentional control under both top-down and bottom-up conditions. Specifically, they examined attentional orienting, the ability to rapidly and accurately direct attention to a target, which is a fundamental component of attentional control. They found that bilinguals were less susceptible to invalid cues and demonstrated faster attentional orienting than monolinguals. This pattern indicates that bilinguals possess more efficient attentional control and monitoring, as they were better able to use cues to guide attention while continuously tracking the predictive value of those cues in the environment. Additionally, the AX-Continuous Performance Task (AX-CPT), a paradigm that measures proactive control by requiring participants to maintain contextual cues over a delay period, has been used to examine proactive monitoring, a key aspect of attentional control, with bilinguals outperforming monolinguals on this task (Beatty-Martínez et al., 2020b; Gullifer et al., 2018; Morales et al., 2013).

To investigate attentional control, the present study employed the anti-saccade task—a well-established measure that engages multiple components of attentional processing (Draheim et al., 2021). In this task, participants must inhibit a reflexive saccade toward a peripheral cue and instead execute a voluntary saccade in the opposite direction. Successful performance requires not only inhibition of the prepotent response, but also sustained goal maintenance (holding the task rule in mind), attentional orienting (directing attention away from the cue), and preparatory set (readiness to execute the correct response; Frischkorn and Oberauer, 2025). Thus, although the anti-saccade task is often classified as a measure of inhibitory control, its demands extend beyond inhibition to encompass the broader construct of attentional control (Engle, 2018). This makes it well-suited for testing hypotheses about bilingual attentional adaptation, as it captures the multifaceted nature of attentional regulation rather than a single, isolated process.

In summary, compared to traditional inhibitory control frameworks (Green, 1998), attentional control theory offers a more integrated perspective for explaining the impact of bilingual experience on cognitive control. Existing research suggests that long-term bilingualism enhances individuals' ability to allocate attentional resources efficiently during multitasking and filter conflicting information, thereby increasing the flexibility and adaptability of cognitive systems. However, most evidence to date comes from correlational studies comparing bilinguals and monolinguals, This leaves open questions about the threshold conditions under which attentional control is engaged. It also remains unclear whether it can be transiently modulated by the intensity and cumulativity of immediate contextual demands.

### 1.3 Current research

The present study systematically tests the above reasoning through two experiments. Experiment 1 used a single naming task to simulate different interactional contexts, aiming to examine whether a single exposure to dual-language contexts would induce any change in attentional control. Experiment 2 employed two consecutive naming tasks to increase contextual exposure, allowing us to compare the effects of single vs. repeated exposure and to explore whether a threshold effect might emerge.

The picture naming task was chosen for two reasons. First, it allows flexible manipulation of language control demands across different interactional contexts. Second, it has been widely used in cross-task priming paradigms to examine language-cognition interactions (Jiao et al., 2020a). A single naming task was used in Experiment 1 to establish whether a minimal level of contextual engagement would produce any observable effect on subsequent attentional control. Two consecutive naming tasks were used in Experiment 2 to increase cumulative cognitive load, allowing us to test whether repeated engagement within the same context type would produce a different pattern of results.

Two consecutive blocks were chosen as an initial step based on the MART framework, which suggests that attentional resources may be dynamically adjusted in response to cumulative task demands. A single exposure allows us to examine whether a minimal level of contextual engagement induces any change in attentional control, whereas two consecutive exposures allow us to explore whether increased cumulative load produces a different pattern of results. This basic comparison provides an initial test of a possible threshold effect while minimizing potential fatigue that might accompany more extended exposure.

The comparative design of the two experiments aims to uncover the underlying mechanisms by which bilingual contexts modulate attentional control. Specifically, whether such effects are dominated by differences in context types or determined by the cumulativity of exposure intensity. Answering this question may provide a novel theoretical perspective for research on bilingual cognitive plasticity.

## 2 Experiment 1: effect of a single interactional context prime on attentional control

To investigate whether the activation of a specific interactional context can transiently modulate domain-general attentional control, Experiment 1 employed a cross-task paradigm. This paradigm involves a task shift, from a language naming task (monolingual, bilingual, or voluntary naming context) to an anti-saccade task (a well-established measure of attentional control). The rationale for employing this task shift is twofold. First, if attentional control is a domain-general capacity shaped by bilingual language experience, its engagement should be observable across task domains, extending beyond language processing to non-linguistic cognitive performance. Second, and more critically, this cross-task design allows for a systematic investigation of the minimum intensity of contextual engagement required to modulate attentional control. A single naming task was chosen as the baseline condition. According to MART, a single exposure to high-demand dual-language contexts may influence attentional resources, but the direction of this effect (depletion, no change, or facilitation) is unknown. Experiment 1 thus examines whether any change occurs at this minimal level, providing a baseline for comparison with repeated exposure in Experiment 2. Through this design, Experiment 1 provides a foundational test of the immediate, short-term influence of interactional context on attentional control.

### 2.1 Participants

A priori sample size estimation was conducted using G^*^Power 3.1 (Faul et al., 2007) for a one-way repeated measures ANOVA with four within-subject conditions (monolingual Chinese, monolingual English, bilingual, and voluntary naming contexts). Based on an assumed medium effect size (*f* = 0.25), α = 0.05, power (1–β) = 0.80, and the default correlation among repeated measures (*r* = 0.5), the analysis indicated a required sample size of 24 participants. Sixty bilingual Chinese–English speakers from Inner Mongolia University of Nationalities were selected for the study (mean age = 19.86 years, *SD* = 1.42; 24 males, 36 females). All participants were right-handed with normal or corrected vision. Their native language was Chinese, and they had passed the College English Test Band 4. All subjects signed informed consent forms. The 10-point Likert scale self-assessment of language proficiency was used to evaluate proficiency in listening, speaking, reading, and writing for both L1 (first language, Chinese) and L2 (second language, English). A score of 1 indicated very low proficiency, while 10 indicated very high proficiency. The mean English proficiency score was 6.43, and the mean Chinese proficiency score was 8.54. The average age of English acquisition was 3.55 years. Subjective socio economic status (SES) was assessed based on parental occupation and education level. Subjective socio economic status (SES) was 5.22, objective SES was 10.31, and the Raven'sA priori sample size estimation was conducted using Progressive Matrices test average was 11.71.

### 2.2 Design

Experiment 1 employed a single-factor within-subjects design. The independent variable interactional context was manipulated across four levels: Chinese monolingual context, English monolingual context, bilingual context, and voluntary naming context. Monolingual contexts comprised Chinese-only picture naming and English-only picture naming. Bilingual contexts involved naming in the corresponding language based on color cues. Voluntary naming contexts adopted a voluntary picture naming paradigm in which participants voluntarily chose to name each picture in either Chinese or English, without any cue.

### 2.3 Materials

The experimental materials consisted of 88 black-and-white line drawings (Zhang and Yang, 2003). The images depicted concepts from common semantic categories, including 11 categories: fruits, tools, clothing, furniture, transportation, poultry, vegetables, toys, body organs, stationery, and mammals. This selection ensured a sufficient number of trials across the four context conditions, with items evenly distributed across categories to minimize category-specific biases and balance stimulus difficulty across conditions.

### 2.4 Procedure

Employing cue-prompted naming tasks to induce monolingual and bilingual contexts (Jiao et al., 2020b; Wu et al., 2020) and employed voluntary naming tasks to simulate the voluntary naming context trials (Beatty-Martínez et al., 2020a; De Bruin et al., 2018). Different interactional contexts were primed using picture naming tasks. After completing picture naming tasks participants performed a anti-saccade task. During the picture naming phase, participants named the images sequentially presented at the center of the screen in Chinese or English based on the color of the prompt box, according to the four levels of interactional context. Chinese monolingual context: All trials presented a red border, and participants named the images in Chinese. English monolingual context: All trials presented a blue border, and participants named the images in English. Bilingual context: Red and blue borders appeared alternately and randomly across trials, and participants named the images in the language indicated by the border color. Voluntary naming context: Red and blue half-frames appeared simultaneously on the screen, with red and blue alternating between top and bottom positions across trials. Participants were free to choose to name each image in either Chinese or English. Following the picture naming trials, the anti-saccade trials presented a star symbol, and participants were required to ignore the star's actual location and look toward its opposite position.

Prior to the formal experiment, participants completed a familiarization phase. They were first shown all 88 pictures with their Chinese and English labels, followed by a second presentation round. Then, they named each picture in both languages without labels. Accuracy was checked separately for Chinese and English. If accuracy in either language fell below 90%, the familiarization procedure was repeated until the threshold was met for both languages. This threshold ensured that all participants had sufficient mastery of the picture names before proceeding to the formal experiment. Subsequently, participants completed practice sessions that resemble the formal experiment, consisting of 32 trials and using eight different images as stimuli. The sequence of Chinese and English presentations remained consistent across the familiarization, practice, and formal experiment phases and was balanced across participants.

The experimental procedure consisted of four blocks, corresponding to four priming conditions: Chinese monolingual context, English monolingual context, bilingual context, and voluntary naming context. Within each block, picture naming trials were followed by anti-saccade trials, totaling 60 picture naming trials and 60 anti-saccade trials. To control for potential order effects while preventing carryover effects from the switching contexts contaminating the monolingual baseline, the presentation order of the four blocks was arranged using a Latin Square design. This design ensured that each of the four context conditions appeared equally often in each ordinal position across participants, while also ensuring that each condition preceded and followed every other condition an equal number of times. The Latin Square yielded four distinct presentation sequences, which were randomly assigned to participants.

**Figure 1:**
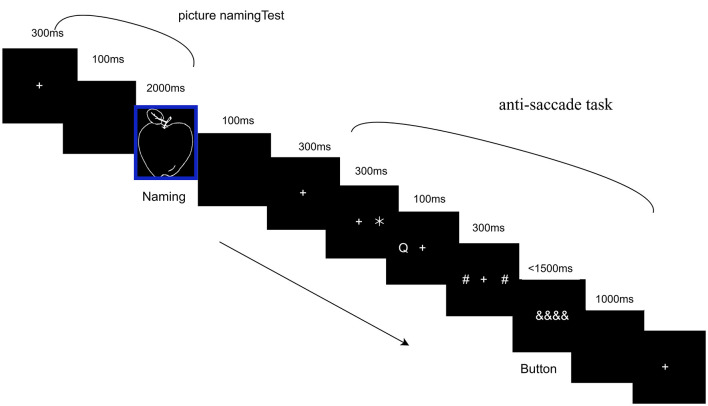
Experiment 1 flowchart. The symbols +, *, Q, #, and &&&& represent the sequence of stimuli presented on the screen during the picture naming and anti-saccade tasks, as described in the Procedure section.

Each trial commenced with a 300 ms fixation point at the screen center, followed by a 100 ms blank screen, then a 2,000 ms image stimulus. Participants were required to identify the image they observed verbally. Following the picture naming trial, a 100 ms blank screen appeared, followed by a 300 ms fixation point. A 300 ms asterisk then appeared at a 12° visual angle to the left or right of the fixation point. Immediately after, the target letter “Q” or “O” appeared for 100 ms on the opposite side of the asterisk. A “#” symbol then masked the positions of both the asterisk and target letter for 300 ms. When “&&&&” appeared on the screen, the participant was required to respond with a keypress within 1,500 ms. The participant's task was to ignore the asterisk and shift their gaze to the opposite side of the screen to search for the target letter “Q” or “O.” If the target was “Q,” press the “Q” key; if the target was “O,” press the “O” key. After a 1,000 ms blank screen, the next trial began, as shown in Figure 1.

### 2.5 Results

Statistical analysis of reaction times and accuracy for the anti-saccade task was conducted using SPSS 27.0. The accuracy rate for the anti-saccade task remained consistently high across all four contextual conditions, averaging 96.50%. Consequently, only reaction time data were analyzed. Data from two participants were excluded due to incomplete task performance, and subsequent analyses were conducted on the remaining 58 participants. Error trials and outliers were removed prior to analysis. Specifically, trials with reaction times shorter than 80 ms or longer than 1,000 ms were excluded (Jianping and Yue, 2013). Subsequently, outlier data exceeding the mean ± 2.5 standard deviations were removed for each condition. In total, 3% of trials were excluded from the analysis. The code-switching rate under the voluntary naming context was 51.28%. All statistical analyses were conducted on these participant-level means, as shown in Table 1.

**Table 1:** Anti-saccade reaction times and accuracy rates (*M* ± *SD*) under different contextual conditions in Experiment 1.

Measure	English monolingual context	Chinese monolingual context	Bilingual Context	Voluntary naming context
Reaction time (ms)	249.99 ± 78.48	246.82 ± 76.30	386.21 ± 76.23	373.58 ± 82.83
Accuracy (%)	0.97 ± 0.03	0.97 ± 0.02	0.97 ± 0.02	0.95 ± 0.12

**Figure 2:**
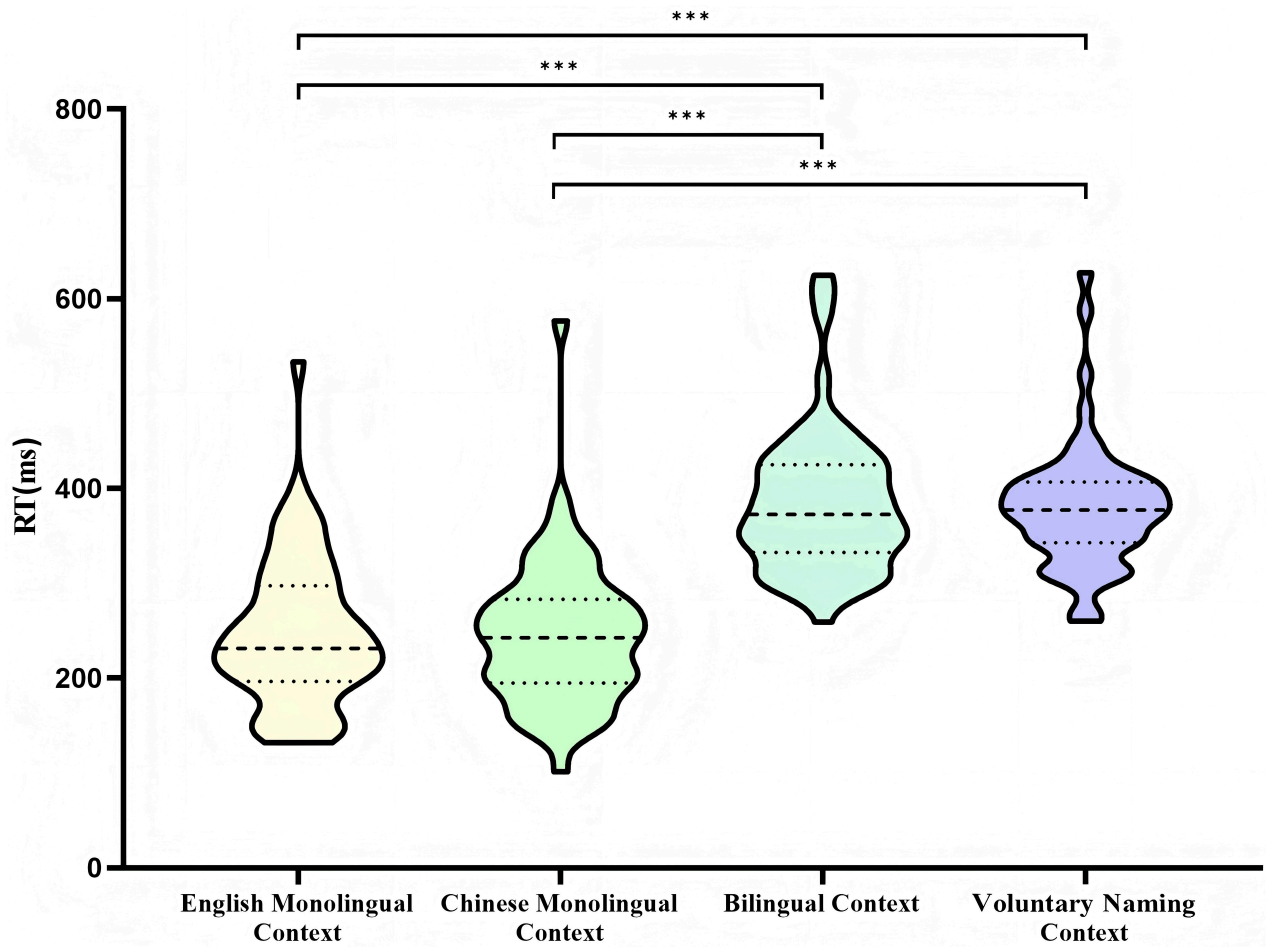
Reaction times (RT) in the anti-saccade task under different context conditions. ^*^p < 0.05, ^**^p < 0.01, ^***^p < 0.001.

The one-way repeated measures ANOVA revealed a significant main effect of Interactional Context on anti-saccade reaction times: *F*_(3, 171)_ = 100.91, *p* < 0.001,$\eta_{p}^{2}$ = 0.639. *Post-hoc* pairwise comparisons were conducted using paired-sample *t*-tests with Bonferroni correction for multiple comparisons. Table 2 showed that reaction times were significantly longer following both dual-language contexts (bilingual and voluntary naming) compared to both monolingual contexts (all *ps* < 0.001). No significant difference was found between the two dual-language contexts (*p* = 0.21). Figure 2 presents the complete set of pairwise comparisons.

**Table 2:** pairwise comparisons of anti-saccade reaction times across context conditions in experiment 1.

Comparison	*t*	*p*	Cohen's *d*
Bilingual vs. English monolingual	13.91^***^	< 0.001	1.826
Bilingual vs. Chinese monolingual	13.31^***^	< 0.001	1.747
Voluntary naming vs. English monolingual	9.58^***^	< 0.001	1.258
Voluntary naming vs. Chinese monolingual	10.22^***^	< 0.001	1.341
Voluntary naming vs. Bilingual	1.27	0.21	0.166

^*^p < 0.05, ^**^p < 0.01, and ^***^p < 0.001.

### 2.6 Discussion

Experiment 1 primarily investigated the effects of different interactional contexts on bilinguals' attentional control, focusing on the immediate impact of single-exposure dual-language engagement. The anti-saccade task accuracy rate was consistently high (*M* = 96.50%) across all four contexts, showing no significant differences; this ceiling effect indicates that the anti-saccade task was relatively easy for participants in terms of response accuracy, and thus the task difficulty was not sufficient to detect potential differences in attentional control accuracy across contexts. In contrast, reaction time data showed significant differences: anti-saccade reaction times were significantly longer following bilingual and voluntary naming contexts relative to monolingual contexts, indicating that single dual-language exposure induced an attentional resource depletion effect rather than facilitating subsequent attentional control.

This interference effect can be well-explained by the Malleable Attentional Resources Theory (Young and Stanton, 2002), which posits that attentional capacity is not a fixed reservoir but a system that dynamically adjusts in response to task demands. Specifically, a single and transient engagement in the high-demand dual-language contexts (bilingual and code-switching) fails to trigger the adaptive expansion of attentional capacity in the cognitive system. Instead, this short-term high cognitive demand directly depletes bilinguals' baseline attentional resources, leaving insufficient resources to support efficient performance on the subsequent anti-saccade task. This pattern is consistent with a resource depletion account derived from MART. Unlike monolingual contexts, which only require basic target language maintenance, bilingual, and voluntary naming contexts impose substantially higher executive demands during picture naming (Green and Abutalebi, 2013). Bilinguals must continuously engage in target language selection, monitor cross-linguistic interference, and inhibit the non-target language—all core language control processes that consume attentional resources (Xue et al., 2026). Crucially, successful performance on the anti-saccade task relies on the coordinated engagement of multiple attentional control components: inhibition of prepotent reflexive responses, sustained goal maintenance, and flexible attentional orienting. The depletion of attentional resources directly impairs these processes, resulting in longer reaction times (Frischkorn and Oberauer, 2025).

The absence of facilitation in Experiment 1 further suggests that single-exposure contextual priming is insufficient to induce a stable attentional readiness state in bilinguals. Brief dual-language engagement only leads to resource consumption. It does not allow the cognitive system to form a transferable attentional control system that can benefit subsequent non-linguistic tasks. This finding aligns with the dynamic plasticity perspective of executive attention (Engle and Kane, 2003), which emphasizes that attentional control modulation requires cumulative, rather than transient, cognitive engagement.

The present study adopted a cross-task priming paradigm similar to that used by Jiao et al. (2020b), who found neural but not behavioral effects of a single exposure to a mixed-language context in unbalanced bilinguals. Extending their findings, the present study showed that a single exposure produced behavioral costs (prolonged reaction times) rather than facilitation. This contrast suggests that neural adaptations may precede behavioral gains, and that cumulative exposure is necessary to translate neural efficiency into observable behavioral benefits. For unbalanced bilinguals, who face greater inherent language control demands due to asymmetric L1/L2 proficiency, single exposure to dual-language contexts may trigger neural adjustment but results in attentional resource exhaustion, supporting the threshold hypothesis.

Thus, Experiment 1 establishes a critical baseline: the cognitive demands of dual-language contexts alone do not guarantee attentional facilitation; rather, the intensity of contextual engagement may constitute a key threshold variable. Experiment 2 intensified contextual priming through two consecutive picture naming tasks. Specifically, we examined whether repeated dual-language engagement could shift the effect from resource depletion to attentional facilitation. We also aimed to clarify the dose-dependent relationship between interactional context exposure and attentional control modulation.

## 3 Experiment 2: effect of repeated interactional context primes on attentional control

To further test the cumulative threshold effect of cognitive load and to address the finding of Experiment 1 (where a single exposure did not induce facilitation), Experiment 2 intensified the contextual priming by using two consecutive picture-naming tasks before the anti-saccade task. Based on the Malleable Attentional Resources Theory (MART), the lack of facilitation in Experiment 1 may indicate that the cognitive load from a single exposure was insufficient to reach the threshold for adaptive reorganization. Therefore, in Experiment 2, we increased contextual exposure to two consecutive blocks. We explored whether repeated engagement would surpass the threshold and trigger adaptive changes in attentional control, unlike the pattern observed in Experiment 1.

### 3.1 Participants

A priori sample size estimation was conducted using G^*^Power 3.1 (Faul et al., 2007) for a one-way repeated measures ANOVA with four within-subject conditions (monolingual Chinese, monolingual English, bilingual, and voluntary naming contexts). Based on an assumed medium effect size (*f* = 0.25), α = 0.05, power (1–β) = 0.80, and the default correlation among repeated measures (*r* = 0.5), the analysis indicated a required sample size of 24 participants. Forty independent bilingual Chinese–English speakers (not overlapping with Experiment 1) from Inner Mongolia University of Nationalities were selected for the experiment (mean age = 19.90 years, *SD* = 1.41; 24 males, 36 females). All participants were right-handed with normal or corrected vision. Their native language was Chinese, and they had passed the College English Test Band 4. All participants signed informed consent forms. Basic participant information was assessed using the same method as Experiment 1. English proficiency was 6.44, and Chinese proficiency was 8.82. The average age of English acquisition was 3.77 years. Subjective socio economic status (SES) was assessed based on parental occupation and education level. Subjective SES was 5.00, objective SES was 11.80, and the average Raven's test score was 13.47. Comparison of background measures between Experiment 1 and Experiment 2 revealed no significant differences (*ps* > 0.14), confirming that the two participant groups were comparable across language proficiency, age of acquisition, SES, and Raven's scores.

### 3.2 Design

Same as Experiment 1.

### 3.3 Materials

Same as Experiment 1.

### 3.4 Procedure

**Figure 3:**
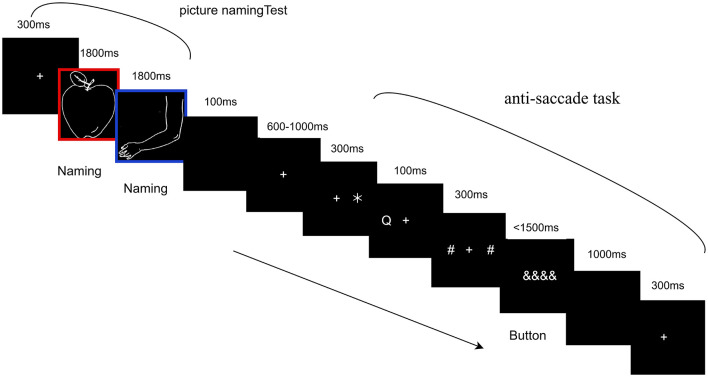
Experiment 2 flowchart. The symbols +, *, Q, #, and &&&& represent the sequence of stimuli presented on the screen during the picture naming and anti-saccade tasks, as described in the Procedure section.

The experimental procedure was consistent with Experiment 1, with the only difference being that the picture naming task was conducted twice consecutively under the same contextual priming (60 trials for each naming task), as shown in Figure 3.

### 3.5 Results

Statistical analysis of reaction times and accuracy for the anti-saccade task was conducted using SPSS 27.0. Error trials and outliers were removed prior to analysis. Specifically, trials with reaction times shorter than 80 ms or longer than 1,000 ms were excluded (Jianping and Yue, 2013). Five participants with more than 25% of data removed were excluded, leaving 35 participants for subsequent analysis. Subsequently, outliers exceeding the mean ± 2.5 standard deviations were removed for each condition. In total, 5% of trials were excluded from the analysis.

All statistical analyses were conducted on these participant-level means, as shown in Table 3.

**Table 3:** Reaction times and accuracy rates (*M* ± *SD*) for the anti-saccade task under different context conditions in Experiment 2.

Measure	English monolingual context	Chinese monolingual context	Bilingual context	Voluntary naming context
Reaction time (ms)	295.08 ± 100.97	290.94 ± 104.95	236.30 ± 80.53	251.10 ± 79.61
Accuracy (%)	0.92 ± 0.11	0.93 ± 0.10	0.95 ± 0.08	0.96 ± 0.09

**Figure 4:**
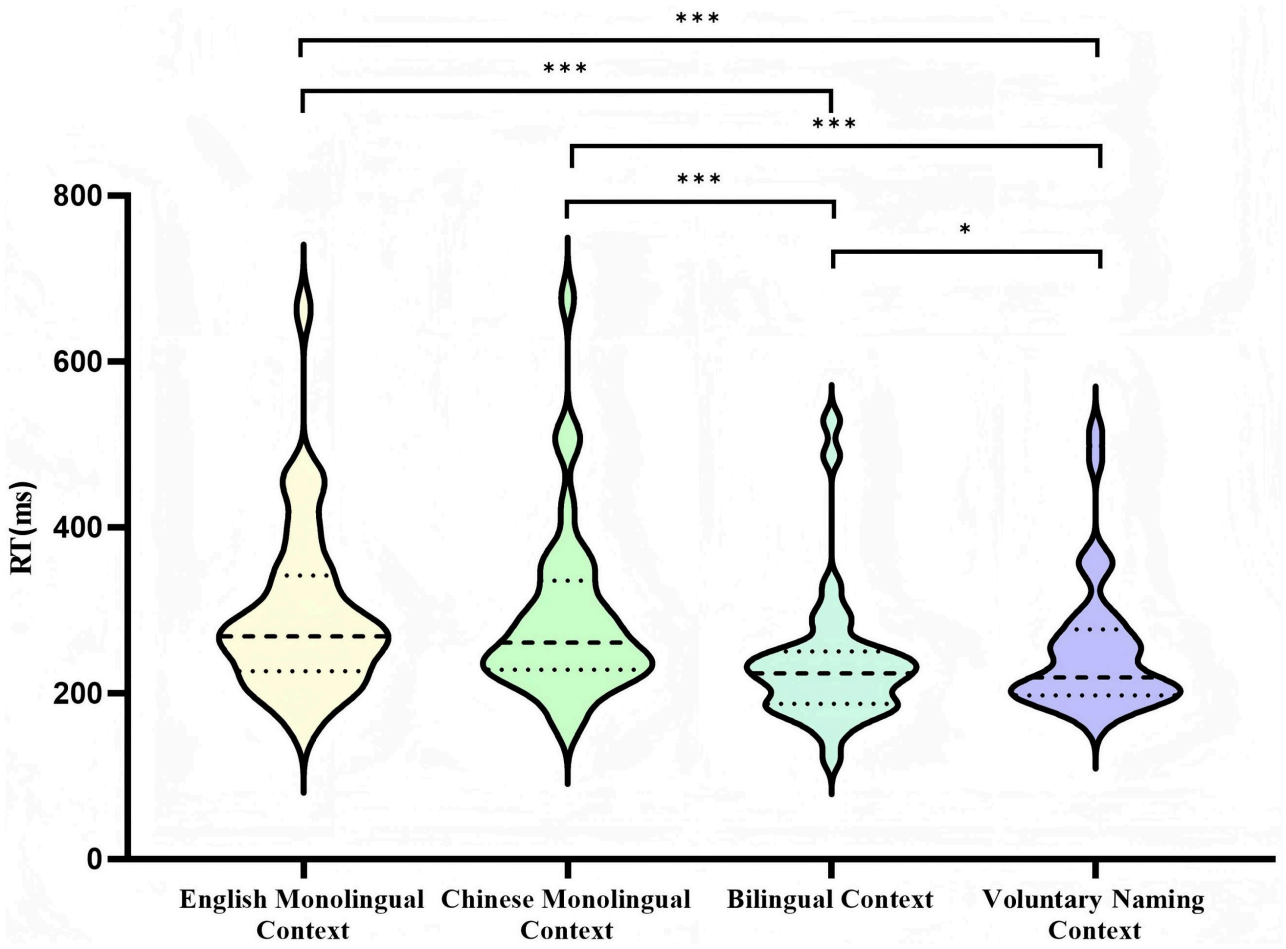
Reaction times (RT) for the anti-saccade task across different context conditions in Experiment 2. ^*^p < 0.05, ^**^p < 0.01, ^***^p < 0.001.

A one-way repeated measures ANOVA on reaction time data revealed a significant main effect of Interactional Context: *F*_(3, 102)_ = 14.377, *p* < 0.001,${\;\eta }_{p}^{2}=$ 0.297. *Post-hoc* pairwise comparisons were conducted using paired-sample *t*-tests with Bonferroni correction for multiple comparisons. Table 4 showed that reaction times were significantly shorter following both dual-language contexts (bilingual and voluntary naming) compared to both monolingual contexts (all *ps* < 0.001). Furthermore, reaction times were significantly shorter following the bilingual context than following the voluntary naming context (*p* = 0.024). Figure 4 presents the complete set of pairwise comparisons.

**Table 4:** Pairwise comparisons of anti-saccade reaction times across context conditions in Experiment 2.

Comparison	*t*	*p*	Cohen's *d*
Bilingual vs. English monolingual	5.46^***^	< 0.001	0.923
Bilingual vs. Chinese monolingual	4.90^***^	< 0.001	0.829
voluntary naming vs. English monolingual	3.57^***^	< 0.001	0.603
voluntary naming vs. Chinese monolingual	4.15^***^	< 0.001	0.702
Bilingual vs. voluntary naming	2.36^*^	0.024	0.399

^*^p < 0.05, ^**^p < 0.01, and ^***^p < 0.001.

**Figure 5:**
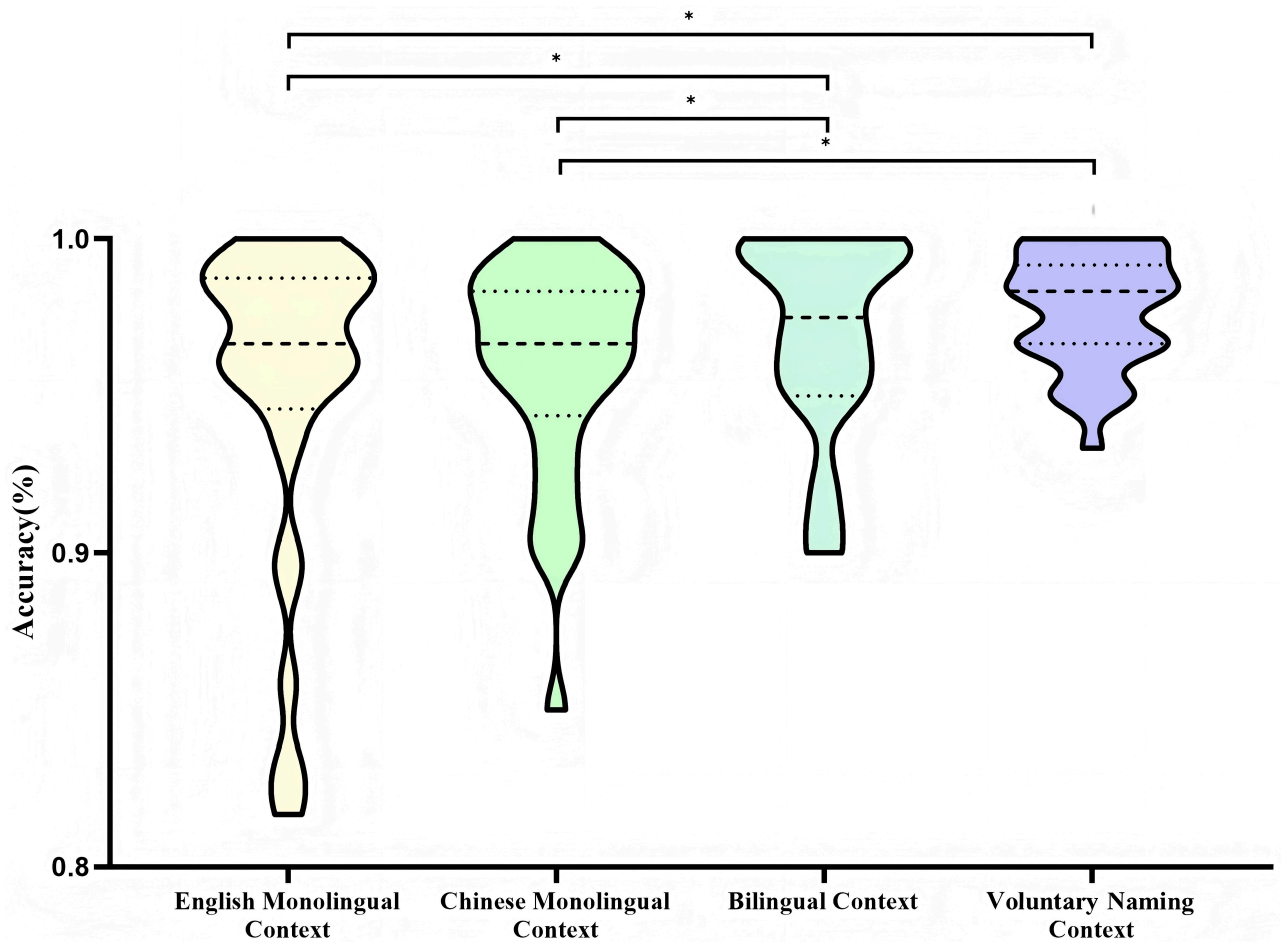
Accuracy rates for the anti-saccade task under different contextual conditions in Experiment 2. ^*^p < 0.05, ^**^p < 0.01, ^***^p < 0.001.

A one-way repeated measures ANOVA on accuracy data revealed a significant main effect of Interactional Context: *F*_(3, 102)_ = 3.85, *p* = 0.012, $\eta_{p\;}^{2}=$ 0.102. *Post-hoc* pairwise comparisons were conducted using paired-sample *t*-tests with Bonferroni correction for multiple comparisons. Table 5 showed that accuracy was significantly higher following both dual-language contexts (bilingual and voluntary naming) compared to both monolingual contexts (all *ps* < 0.05). No significant difference was found between the two dual-language contexts (*p* = 0.77). Figure 5 presents the complete set of pairwise comparisons.

**Table 5:** Pairwise comparisons of anti-saccade accuracy across context conditions in Experiment 2.

Comparison	*t*	*p*	Cohen's *d*
Bilingual vs. English monolingual	2.33^*^	0.03	0.393
Bilingual vs. Chinese monolingual	2.35^*^	0.03	0.393
voluntary naming vs. English monolingual	2.22^*^	0.03	0.374
voluntary naming vs. Chinese monolingual	2.42^*^	0.02	0.374
voluntary naming vs. Bilingual	0.30	0.77	0.050

^*^p < 0.05, ^**^p < 0.01, and ^***^p < 0.001.

### 3.6 Discussion

Experiment 2 employed a two-step naming procedure to strengthen context priming, examining whether repeated dual-language engagement would facilitate attentional control. Results showed clear facilitation: relative to monolingual contexts, both dual-language contexts reduced anti-saccade reaction times and improved accuracy. Moreover, reaction times were shorter in the bilingual context than in the voluntary naming context, suggesting repeated dual-language engagement without frequent switching mobilizes attentional resources more efficiently.

The shift from interference (Experiment 1) to facilitation (Experiment 2) can be interpreted through the cumulative threshold framework dynamic allocation mechanism of attentional resources (Young and Stanton, 2002) and the Adaptive Control Hypothesis (Green and Abutalebi, 2013). For unbalanced bilinguals, brief dual-language exposure (Experiment 1) depletes attentional resources due to the high demands of initial language control. In contrast, repeated dual-language engagement (Experiment 2) allows the cognitive system to form a stable task set for language control. Repeated practice in target selection, interference inhibition, and resource allocation strengthens the activation of domain-general attentional control mechanisms (Jiao et al., 2019). This enables efficient mobilization and reallocation of attentional resources rather than mere consumption. This stable task set transfers to the subsequent anti-saccade task, enhancing performance across multiple attentional control components (inhibition, orienting, and goal maintenance) and resulting in shorter reaction times and higher accuracy.

The finding that the bilingual context produced a stronger facilitatory effect than the voluntary naming context aligns with the prediction that the nature of cognitive load differs across dual-language contexts (Green and Abutalebi, 2013). Bilingual contexts require sustained goal maintenance, interference suppression, and cue monitoring—demands that directly engage and train the attentional control system. Voluntary naming contexts, although also cognitively demanding, involve opportunistic planning and real-time switching decisions that partially circumvent competitive inhibition. In other words, the cognitive load in voluntary naming contexts is more oriented toward language integration rather than inhibitory control, making it less effective in mobilizing the attentional control system for subsequent non-linguistic tasks. This interpretation is consistent with prior research showing that different interactional contexts impose distinct linguistic control demands, which in turn differentially modulate cognitive control outcomes (Beatty-Martínez et al., 2020b; Hartanto and Yang, 2020).

Collectively, these results confirm that repeated exposure to dual-language contexts can surpass the cognitive threshold necessary to trigger adaptive reorganization of attentional control— a pattern consistent with the gain effect predicted by the threshold framework. The stronger facilitation observed in the bilingual context further supports the notion that the qualitative nature of cognitive load (suppression-oriented vs. integration-oriented) moderates the extent to which repeated engagement translates into attentional gains.

These findings extend the framework of Bialystok and Craik (2022) on bilingual attentional control modulation, demonstrating that the cognitive effects of bilingual experience are state-dependent and context-sensitive (Bialystok and Craik, 2022). Bilingual-induced attentional control changes are not limited to lifelong trait-like advantages from long-term language use; transient but repeated engagement with dual-language contexts can rapidly induce adaptive changes in the attentional control system. This highlights the dynamic plasticity of attentional control (Engle and Kane, 2003) in bilinguals: the system can adjust its processing efficiency in response to the intensity of immediate contextual cognitive demands. For unbalanced bilinguals, who bear higher daily language control demands and thus may have greater potential for bilingual cognitive advantages (Mouthon et al., 2020), repeated dual-language engagement acts as a key trigger to activate this plasticity and convert potential advantages into actual attentional control improvement.

In summary, Experiment 2 complements Experiment 1 by verifying the threshold relationship between dual-language context exposure and attentional control modulation: repeated engagement crosses the cognitive threshold from resource depletion to adaptive activation, leading to attentional facilitation. This two-experiment design provides clear experimental evidence for the cumulative effect of interactional context on bilingual attentional control, laying the foundation for a comprehensive theoretical discussion of the threshold hypothesis and its underlying cognitive mechanisms.

Consistent with the cumulative threshold framework, surpassing the cognitive load threshold in Experiment 2 enhanced multiple components of attentional control: reaction time improvements reflected faster attentional orienting; accuracy improvements reflected better goal maintenance and inhibitory control.

## 4 General discussion

This study, through two consecutive cross-task priming experiments, revealed a threshold effect in the influence of bilingual interactional contexts on attentional control: one block of exposure to a dual-language context (60 picture naming trials) fell below the cognitive load threshold, depleting attentional resources and impairing anti-saccade performance; two consecutive blocks of exposure (120 trials in total) surpassed the threshold, triggering an adaptive enhancement of attentional control. Contexts with clear language boundaries produced a stronger facilitatory effect than voluntary naming contexts. This finding advances bilingual cognition research from static context-type comparisons toward a dynamic, intensity-dependent framework.

In summary, the main pattern of effects is as follows: (1) a single exposure to dual-language contexts led to resource depletion (longer reaction times); (2) repeated exposure to dual-language contexts led to attentional facilitation (shorter reaction times and higher accuracy); (3) the bilingual context produced stronger facilitation than the voluntary naming context. These findings collectively support a cumulative threshold framework, wherein the intensity and cumulativity of contextual exposure determine whether attentional control is depleted or enhanced.

### 4.1 From resource depletion to cognitive gain

The contrast between Experiment 1 and Experiment 2 clearly reveals a cumulative threshold effect of cognitive load. A single exposure to dual-language contexts (Experiment 1) produced only resource depletion, manifested as prolonged anti-saccade reaction times; in contrast, two consecutive exposures (Experiment 2) transformed into a facilitatory effect, reflected in shorter reaction times and higher accuracy. This transition cannot be explained by traditional bilingual advantage or interference accounts. Instead, it aligns with the core prediction of the Malleable Attentional Resources Theory (Young and Stanton, 2002). The theory proposes that the dynamic regulation of attentional resources follows a threshold principle. Adaptive reorganization occurs only when cumulative load exceeds the system's current regulatory capacity.

In Experiment 1, the high instantaneous load of a single naming task fell below the threshold: language control processes extensively consumed resources without triggering reorganization, resulting in insufficient resources for the subsequent task. In Experiment 2, two consecutive naming tasks allowed load to accumulate above the threshold, forcing the attentional control system to shift from passive consumption to active reorganization, thereby forming a transferable attentional control system. This interpretation is supported by neurophysiological evidence: sustained cognitive demands can enhance prefrontal cortex functional connectivity and lower the recruitment threshold of control mechanisms (Huang et al., 2024). The present study provides convergent behavioral evidence for this view.

### 4.2 Context type modulates the threshold effect

Under repeated exposure conditions, the facilitatory effect was significantly stronger for the bilingual context than for the voluntary naming context. This difference can be understood in terms of the qualitative nature of cognitive load in each context. According to the Adaptive Control Hypothesis (Green and Abutalebi, 2013), bilingual contexts require separate language use, with load concentrated on goal maintenance and non-target suppression—demands that are focused and consistent, facilitating the formation of stable task-set representations. When consecutively exposed to the same context, this task set is repeatedly activated and strengthened. Once strengthened, the adapted attentional control system enhances subsequent anti-saccade performance.

In contrast, voluntary naming contexts allow voluntary language mixing, imposing more complex and heterogeneous load. Beyond goal maintenance and interference suppression, they add the extra demands of real-time switching decisions and linguistic structure integration (Beatty-Martínez et al., 2020a). Although, these additional demands also consume resources, the voluntary naming context follows an opportunistic planning principle (Beatty-Martínez et al., 2020a), which favors language integration over sustained suppression. Thus, its cognitive load is less focused and less intense than that of the bilingual context. A portion of cognitive resources must be continuously devoted to online monitoring rather than fully invested in task-set consolidation. Consequently, even if the total accumulated load may be higher, the proportion of resources available for effective reorganization is lower than in the bilingual context. The reaction time data support this interpretation: anti-saccade responses were significantly faster following the bilingual context than the voluntary naming context, indicating that the former's attentional control system exhibited greater transfer efficiency. Accuracy data further show that both contexts successfully triggered adaptive enhancement, but with different degrees of efficiency.

### 4.3 From type comparisons to an intensity-dependent framework

The core theoretical contribution of this study lies in demonstrating, that identical context types can produce opposite effects depending on exposure intensity. This finding reveals a hitherto overlooked variable—the cumulativity of contextual engagement—that plays a critical role in bilingual cognitive plasticity. Previous research has largely focused on the differential effects of context types on cognitive control (Hartanto and Yang, 2020; Jiao et al., 2020a). However, the present study shows that even when context type is held constant, cognitive gains remain elusive if exposure intensity fails to reach the threshold. This offers a novel explanatory mechanism for the heterogeneity of proposed bilingual cognitive advantages: why do some studies fail to find advantages? Possibly because participants' daily cumulative language load falls below their individual regulatory threshold. Why are advantages more pronounced in certain tasks? Possibly because those tasks' attentional control demands better match the nature of the contextual load.

Moreover, the threshold concept adds a temporal dimension to the Adaptive Control Hypothesis. While the original framework focuses on how context types differentially demand cognitive control, the present study demonstrates that duration and repetition of exposure are equally critical: only sustained, repeated exposure can translate potential demands into actual plastic changes. This view resonates with the Bilingual Experience Trajectory Model (DeLuca et al., 2020), which emphasizes the multidimensional and continuous nature of bilingual experience.

### 4.4 Automatic adaptation not strategic adoption

A key theoretical question raised by the observed facilitation effect is whether it stems from implicit, automatic adaptive changes in the attentional control system or conscious strategic adoption by participants. Multiple lines of evidence from the experimental design and results collectively support the automatic adaptation account, ruling out conscious strategy as the primary driver of the facilitation effect.

First, the cross-task paradigm minimized the possibility of strategic transfer between tasks. The picture naming task (linguistic) and anti-saccade task (non-linguistic) are independent in task nature and processing requirements. No explicit instruction was given for participants to transfer strategies or processing methods between the two tasks (Jiao et al., 2020a).

Second, the differential effects of bilingual and voluntary naming contexts further challenge a strategic interpretation. If participants had adopted a general-purpose cognitive strategy, it should have benefited both dual-language contexts equally. Yet, the facilitation effect was significantly stronger for the bilingual context than for the voluntary naming context, indicating that the effect is sensitive to the specific nature of contextual demands rather than driven by a one-size-fits-all strategy.

Third, the concurrent improvement in both reaction time and accuracy rules out speed-accuracy trade-off as an alternative explanation and provides further evidence for genuine enhancement of cognitive efficiency—a signature of automatic adaptation rather than conscious strategy use (Draheim et al., 2022). The stable performance across conditions (no practice effect) additionally excludes strategic strengthening, as conscious strategies would be expected to improve with repeated task completion.

Therefore, the threshold effect observed in this study more likely reflects implicit, use-dependent plasticity in the attentional control system—a form of adaptation that operates without conscious effort and is driven by cumulative engagement rather than momentary strategic decisions. This conclusion aligns with the recent theoretical shift in bilingual cognition research (Bialystok and Craik, 2022), which emphasizes that bilingual experience shapes cognition not by “training” specific abilities but by reshaping the overall operating efficiency of the attentional control system.

### 4.5 Bilingualism as an ideal model for studying cognitive engagement and plasticity

A core controversy in bilingual cognitive research is whether the cognitive effects of bilingual experience are unique, given that non-linguistic experiences (e.g., video game playing, musical training, and physical exercise) can also improve executive functions (Friedman and Miyake, 2017). The present study addresses this controversy by clarifying the core value of bilingual interactional context research, rather than arguing for the uniqueness of the bilingual effect.

The results of this study do not suggest that only bilingual experience can modulate attentional control; instead, they confirm that bilingual interactional contexts, as a type of cognitive activity requiring sustained attentional investment, can effectively regulate the attentional control system in accordance with the predictions of the Adaptive Control Hypothesis (Green and Abutalebi, 2013). The unique value of bilingual interactional contexts lies in their high ecological validity and natural occurrence. Language use is a ubiquitous cognitive activity in human daily life, embedded in social interaction. Its cognitive demands (e.g., language control, resource allocation) are continuous and variable (DeLuca et al., 2020). Unlike artificial laboratory cognitive training or specific non-linguistic experiences (e.g., video games), bilingual use is a natural, long-term cognitive engagement that is highly representative of real-world cognitive activity, making it an ideal model for exploring how sustained cognitive demands shape the attentional control system (Bialystok and Craik, 2022).

Furthermore, the threshold pattern observed in this study (resource depletion following a single exposure, facilitation from repeated exposure) is highly consistent with the actual situation of natural bilingual language use: occasional code-switching or brief dual-language engagement in daily life is unlikely to bring about lasting attentional control changes, while long-term and sustained daily bilingual use (e.g., in bilingual communities or immersion environments) can trigger cumulative cognitive adaptation (Deluca et al., 2019). The two-experiment design of the present study is a micro-simulation of this natural cumulative effect, which verifies that laboratory-based contextual priming can effectively capture the core law of bilingual cognitive adaptation—cumulative engagement is the key to triggering attentional control facilitation. This bridge between laboratory manipulation and real-world bilingual experience addresses the criticism that prior bilingual cognitive research is overly divorced from natural language use (Hartanto and Yang, 2020), and provides a feasible experimental paradigm for future research on bilingual cognitive plasticity.

In addition, the finding that linguistic contexts can prime non-linguistic attentional task performance further confirms that bilingual processing activates domain-general attentional control mechanisms (Draheim et al., 2021), which are the same as those invoked by non-linguistic experiences such as video games and musical training. This consistency in underlying mechanisms reveals the existence of a set of common cognitive processes that can be trained and modulated by various sustained cognitive experiences.

### 4.6 Theoretical implications

Centering attentional control in bilingual cognitive adaptation clarifies how bilingual experience shapes cognition. Findings align with the adaptive control hypothesis (Green and Abutalebi, 2013), which links contextual demands to executive function regulation, but extend it in three novel ways.

First, contextual adjustments are cumulative and dose-dependent: they require repeated engagement to be measurable, with magnitude scaling with contextual demand. This extends the hypothesis by specifying not only which contexts matter but also the engagement intensity needed for effects. Second, transient attentional control modulation via experimental manipulation bridges correlational lifelong bilingual studies and laboratory cognitive control research. The threshold pattern suggests bilingual cognitive effects are gradual (not all-or-nothing), emerging via repeated demanding language engagement.

Third, findings contribute to conversations on attentional control, working memory, and intelligence. Engle and Kane (2003) link executive attention (goal maintenance amid interference) to working memory and fluid intelligence. Sustained bilingual exposure may enhance working memory and intelligence indirectly, by first improving attentional control. Attentional control is a foundational component of both working memory and fluid intelligence. Therefore, any enhancement of attentional control (as observed in Experiment 2) should, over time, lead to improvements in these higher-order abilities. critical for understanding real-world experience-driven cognitive change.

In conclusion, interactional contexts exert rapid, cumulative, reversible effects on bilinguals' attentional control—reflecting dynamic domain-general adaptation, not fixed traits or strategies. The threshold framework provides a preliminary mechanistic account of how language experience may shape executive function, potentially resolving literature inconsistencies, extending key theories, and highlighting bilingualism's value as a model for experience-dependent cognitive plasticity.

### 4.7 Limitations

Several limitations of the present study should be acknowledged, which also point to directions for future research.

First, while the within-subjects design controls for stable individual differences, the sample consisted entirely of unbalanced English-Chinese bilinguals. The extent to which these findings generalize to balanced bilinguals or bilinguals with different language pairs remains to be established. Future research should examine whether the threshold for attentional engagement varies across diverse bilingual populations. Additionally, language proficiency was assessed only through self-report measures (10-point Likert scales), which were not standardized or formally validated. No objective English proficiency tests (e.g., LexTALE, Oxford Placement Test) were administered. This limitation should be considered when interpreting participant comparability, as self-reported proficiency may not fully capture actual language ability. Future research should include objective proficiency measures to complement self-report data.

Second, the present study examined only the immediate effects of contextual priming. The duration of the threshold effect remains unknown—can the observed cognitive gains persist for minutes, hours, or longer? Can they accumulate into long-term advantages through repeated training sessions? Addressing these questions requires the introduction of delayed post-tests or longitudinal designs.

Third, the sources of individual differences in the threshold effect (e.g., working memory capacity, language proficiency, and daily language mixing intensity) have not been systematically explored. Future research could employ individual differences designs combined with multidimensional measures of language experience to establish predictive models of the threshold.

Finally, the present study manipulated exposure intensity at only two levels (one block vs. two blocks of picture naming). The dose-response relationship between contextual exposure and attentional control remains to be fully mapped. Future research should systematically vary the number of exposure blocks (e.g., three, four, or more blocks) to examine whether the facilitatory effect continues to increase, plateaus, or reverses with overtraining. Additionally, direct neurophysiological evidence for the threshold effect is needed. Can EEG or fMRI capture plasticity changes in prefrontal networks that correspond to the behavioral threshold pattern? Such evidence would provide a more robust neural foundation for the behavioral findings presented here.

## 5 Conclusion

By comparing single vs. repeated exposure to dual-language contexts, the present study reveals a threshold effect in the influence of bilingual interactional contexts on attentional control: a single exposure fell below the threshold, inducing resource depletion; repeated exposure surpassed the threshold, triggering attentional facilitation. The bilingual context, characterized by clear language boundaries and focused cognitive load, produced a stronger facilitatory effect than the voluntary naming context. Together, these findings make a novel contribution by shifting the focus from static comparisons of context types to a dynamic, intensity-dependent framework of bilingual cognitive adaptation, offering a mechanistic account of how cumulative language experience shapes attentional control.

## Data Availability

The datasets presented in this study can be found in online repositories. The names of the repository/repositories and accession number(s) can be found below: https://pan.baidu.com/s/1XzYTNLeuL6bbr-QnVUtD4Q using the access code 7070.
